# New Tools and Nuanced Interventions to Accelerate Achievement of the 2030 Roadmap for Neglected Tropical Diseases

**DOI:** 10.1093/cid/ciae070

**Published:** 2024-04-25

**Authors:** Andreia Vasconcelos, Cláudio Nunes-Alves, T Déirdre Hollingsworth

**Affiliations:** Big Data Institute, Li Ka Shing Centre for Health Information and Discovery, University of Oxford, Oxford, United Kingdom; Centre for Global Health Research, Nuffield Department of Medicine, University of Oxford, Old Road Campus, Oxford, United Kingdom; Big Data Institute, Li Ka Shing Centre for Health Information and Discovery, University of Oxford, Oxford, United Kingdom

**Keywords:** neglected tropical diseases, mathematical models, elimination, control, policy-making

## Abstract

The World Health Organization roadmap for neglected tropical diseases (NTDs) sets out ambitious targets for disease control and elimination by 2030, including 90% fewer people requiring interventions against NTDs and the elimination of at least 1 NTD in 100 countries. Mathematical models are an important tool for understanding NTD dynamics, optimizing interventions, assessing the efficacy of new tools, and estimating the economic costs associated with control programs. As NTD control shifts to increased country ownership and programs progress toward disease elimination, tailored models that better incorporate local context and can help to address questions that are important for decision-making at the national level are gaining importance. In this introduction to the supplement, New Tools and Nuanced Interventions to Accelerate Achievement of the 2030 Roadmap for Neglected Tropical Diseases, we discuss current challenges in generating more locally relevant models and summarize how the articles in this supplement present novel ways in which NTD modeling can help to accelerate achievement and sustainability of the 2030 targets.

Neglected tropical diseases (NTDs) are a group of 21 conditions caused by a range of bacteria, fungi, parasites, viruses, and toxins that affect more than 1 billion people worldwide [1]. The epidemiology of NTDs is diverse and complex, often characterized by intricate pathogen life cycles, vector-borne transmission and/or the involvement of intermediary hosts, and dependence on environmental conditions, all of which present substantial challenges to disease control and elimination [2]. Despite this heterogeneity, NTDs share common features in their geographical distribution and socioeconomic impact. Affecting predominantly tropical regions worldwide, 80% of the NTD burden is concentrated in just 16 countries [1]. Furthermore, these diseases disproportionately affect resource-poor communities, where they further accentuate economic hardship and perpetuate cycles of poverty.

Over the past decade, significant progress has been made in the control, elimination, and eradication of these diseases, driven by the 2012–2020 World Health Organization (WHO) roadmap for accelerating work to overcome the global impact of NTDs [3]. Notably, the number of people who require NTD interventions globally decreased by 600 million between 2010 and 2020, and 42 countries, areas, and territories successfully eliminated at least 1 NTD [1]. However, many of the 2020 targets were not achieved, prompting WHO to launch the 2021–2030 roadmap that revises disease-specific targets and outlines 3 pillars deemed essential for achieving them: accelerating programmatic action, intensifying cross-cutting approaches, and facilitating country ownership through changes in operating models and culture [4]. Importantly, the 2030 roadmap also aligns NTD targets with the United Nations’ Sustainable Development Goals, representing a step change in policy recognition toward the importance of eliminating these diseases.

To achieve the 2030 targets, national NTD programs require relevant and accurate information that can effectively guide decision-making. This is becoming increasingly important as national health ministries are faced with diverse challenges, such as locally increased transmission rates that resulted from program interruptions due to the coronavirus disease 2019 (COVID-19) pandemic, donor funding cuts, reduced domestic revenues, climate change, and, in some cases, geopolitical instability. In this context, quantitative analysis and epidemiological and economic modeling are important tools for better understanding and forecasting the dynamics of NTDs, informing the design of optimally effective intervention and surveillance strategies, and facilitating the evaluation and implementation of new tools. For example, the NTD modeling community was consulted during the formulation of the 2030 targets [5] and, more recently, partnered with WHO to estimate how disruptions due to the COVID-19 pandemic impacted NTD programs and assess how remedial strategies could help program recovery [6, 7].

Despite these successes, the NTD modeling community is facing new challenges. As the 2021–2030 roadmap emphasizes a push toward country ownership of NTD programs, there is a growing need for models to incorporate local context and focus on priority questions that are most relevant for informing decisions of national and subnational programs [8]. While these priority questions vary across disease and location, they can be summarized into 3 main areas. The first is in understanding how best to achieve the 2030 targets. Here, models are important to determine whether programs are on track and to explore nuanced interventions and/or new tools that can accelerate progress to achieve different end points: disease control, elimination of transmission, elimination as a public health problem (EPHP), or eradication. The second area is in planning for what to do once targets are achieved. The continued success of control programs means that there is a growing emphasis on planning the next stages, focusing on eliminating disease in low-prevalence settings and designing post-validation surveillance strategies to ensure the sustainability of NTD programs and avoid disease resurgence [8]. Models can provide insights into the optimal strategies for stopping interventions and designing monitoring and surveillance after disease elimination. Importantly, such decisions have economic implications due to the need to balance the costs involved in monitoring and surveillance with the benefits of maintaining NTDs under control and avoiding resurgence. This is the third priority area where modeling can help inform decision-making by better incorporating economic parameters.

Here, we focus on these issues by discussing the challenges associated with the transition to more locally relevant models with a stronger emphasis on the final stages of elimination and surveillance. We also summarize how the studies in this supplement demonstrate the ability of NTD models to aid in the design of nuanced interventions tailored to specific contexts and the use of new tools to accelerate achievement of the 2030 targets (Figure 1).

**Figure 1.**
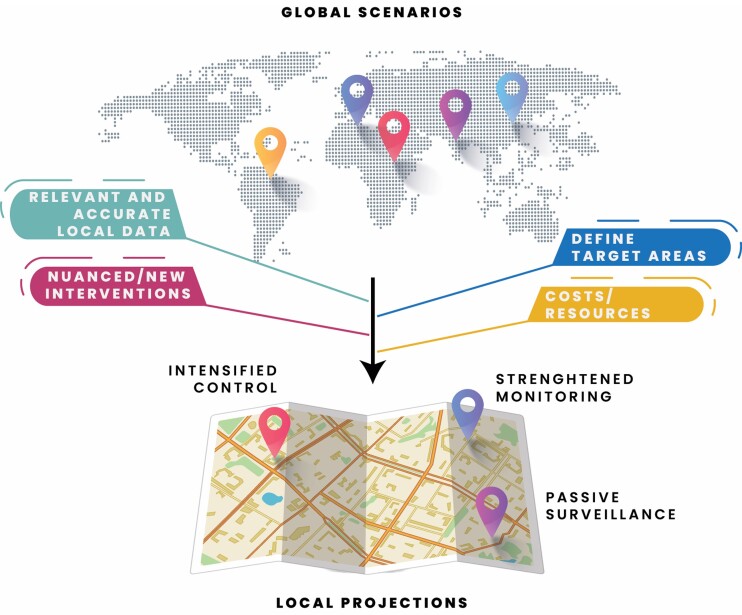
Moving from global scenarios to local projections. Schematic representation of the main concepts discussed, including how models with increased geographical resolution and incorporating local data, information on costs and resources, and considerations of new and nuanced interventions can support programmatic decisions. Credits: *Neighborhood image (below) by Freepik, global map (above): image by macrovector on Freepik*.

## NUANCED INTERVENTIONS AND NEW TOOLS

A crucial way in which NTD models can help accelerate progress toward the 2030 targets is by estimating the likely impact of nuanced or new interventions on disease elimination. For example, models can be used to estimate the likely impact of different numbers of treatment rounds, increasing or expanding coverage, reaching “never-treated” individuals, or deploying new interventions, such as new drugs or vaccines.

### Extending Coverage

Models can be useful in informing decisions on whether to extend current interventions to additional groups. For example, while schistosomiasis interventions have primarily focused on school-aged children (SAC), new guidelines recommend that treatment be expanded to include preschool-aged children (pre-SAC), women of reproductive age, and adults [9]. Individual-based stochastic models that simulate the impact of mass drug administration (MDA) and include these additional groups can help estimate the number of treatment rounds required to meet the 2030 schistosomiasis target of EPHP [10]. These scenario-based models reveal how the number of rounds needed to achieve EPHP depends on the baseline disease prevalence and the treatment coverage used. For example, EPHP can be achieved within 7 years in low-, medium-, and high-transmission areas. However, reaching the entire affected population is challenging for MDA programs, and so the modeling suggests that these results can only be achieved if the percentage of never-treated individuals (ie, those individuals who self-report that they have never ingested tablets during any round of MDA) [11] in these areas is less than 10%, 5%, and 1%, respectively. Therefore, the higher the intensity of transmission and the lower the treatment coverage, the lower the acceptable value of “never treatment” becomes, highlighting how efforts to increase coverage and/or minimize never treatment can shorten program duration.

The proportion of never treatment during MDA campaigns is also a key barrier to the elimination of lymphatic filariasis. Individual-based stochastic models of lymphatic filariasis transmission can be used to estimate the maximum level of never treatment for which lymphatic filariasis targets can be achieved within 10 years under different scenarios (with varying annual MDA coverage, drug combinations, and transmission settings) [12]. These models show that the proportion of never treatment has a strong impact on the achievement of elimination, which is greater in high-transmission areas, and for campaigns based on the use of ivermectin + albendazole (IA). For example, in *Anopheles* transmission settings where the baseline microfilaremia (mf) prevalence is 10%, treating 80% of the eligible population annually with IA can achieve the elimination threshold within 10 years of annual treatment as long as never treatment is lower than 10%. Higher proportions of never treatment are acceptable when lower baseline prevalence and/or more efficacious treatment regimens (such as diethylcarbamazine + albendazole [DA] or ivermectin + diethylcarbamazine + albendazole [IDA]) are used.

### Identifying Target Areas

Another potential use for models is to aid in the identification of areas that may require enhanced treatment to meet WHO elimination criteria. For example, available survey data (on the trachomatous inflammation–follicular [TF] prevalence in children aged 1–9 years) can be used to build an ensemble of probabilistic models to forecast the prevalence of clinical trachoma across 11 760 districts in trachoma-endemic countries [13]. These models identified 172 districts that are likely to exceed the 5% TF control threshold in 2030 with the current interventions, suggesting that global EPHP of trachoma by 2030 may require enhanced intervention and/or surveillance of high-risk districts.

Similar efforts to account for spatial heterogeneities can help achieve the lymphatic filariasis goal of validating EPHP in 58 (81%) of the currently endemic countries, a challenging target particularly for programs in resource-limited countries. For this, a combination of disease transmission models with geospatial statistical modeling can help to estimate the expected progress toward the 2030 goals across 44 sub-Saharan African countries at a fine spatial scale [14]. The resulting projections suggest that although >80% of the endemic countries in Africa are on track to achieve EPHP, pockets of highly endemic locations are likely to miss this target unless they increase the frequency and/or coverage of current interventions.

This combination of geostatistical mapping with transmission modeling can also be useful in elucidating the progress toward lymphatic filariasis elimination at the subnational level, as demonstrated for Ethiopia [15]. While mainly serving as a proof-of-concept example based on historic data from the successful lymphatic filariasis program in Ethiopia, this analysis demonstrates that similar strategies could be implemented in other countries at earlier stages of their lymphatic filariasis programs to help identify areas where elimination is likely to be challenging, so that additional resources can be put in place to accelerate progress.

## TRANSITIONING TO MONITORING AND SURVEILLANCE

Once programs achieve their targets, emphasis needs to shift from how best to achieve elimination to how to optimally wind down interventions and move to post-validation monitoring and surveillance. Such decisions are usually based on predefined prevalence thresholds, as is the case for the stop-MDA decision in lymphatic filariasis programs. MDA of antifilarial drugs is usually based on 2-drug combinations (IA or DA), with stop-MDA decisions for these regimes depending on the prevalence of both mf and circulating filarial antigenemia. However, recent studies showing that the triple drug combination is more effective than dual drug combinations have accelerated the use of IDA for elimination of lymphatic filariasis. As antigen can persist for a long time in people treated with IDA and adults are most likely to be infected and usually participate in MDA at a lower rate than younger age groups, WHO intends to base the stop-MDA decision for IDA-treated areas on mf prevalence in adults. Modeling can help to assess how the probability of reaching elimination depends on this critical threshold used in transmission assessment surveys (TASs) to determine whether transmission was successfully suppressed and whether triple-drug MDA can be stopped [16]. Based on analyses in treatment-naive Indian settings, a single TAS 1 year after the last MDA round provides limited predictive value of having achieved suppressed transmission, while additional surveys conducted in later years (3 and 5 years post-MDA) provide further insights on the prospect of elimination. For example, the predictive value of a single TAS is always <95% for threshold values ≥0.5% mf prevalence, whereas with 2 additional TASs, predictive values of ≥95% are possible for the same threshold, even when MDA coverage is as low as 65%. Therefore, such studies can provide important insights into the best strategies for post-MDA surveillance.

Models can also be used to improve the design of elimination surveys, as in the case of soil-transmitted helminths (STHs). Control of these infections involves the delivery of preventive chemotherapy to SAC through schools. Progress of STH control programs is currently monitored using periodical school-based prevalence surveys, known as impact assessment surveys (IASs). IASs are typically carried out after 5 years of preventive chemotherapy delivery. When the prevalence of STH in the target population falls below 2%, WHO recommends suspending preventive chemotherapy. Given the high cost associated with conducting an IAS, it is important that such surveys are optimally designed and enable the identification of disease hot spots that can be targeted for preventive chemotherapy delivery. Using prevalence data collected in Kenya, the integration of geostatistical methods with a Markov model or a mechanistic transmission model could be used for forecasting STH prevalence, although prediction accuracy was lower in areas with high prevalence hot spots [17].

Insights from modeling can also help to optimize surveillance strategies, including those based on passive surveillance. Using a generic model of disease transmission with slow epidemic growth rates and cases detected through severe symptoms and passive detection, it is possible to identify scenarios under which passive surveillance is sufficient to control disease transmission [18]. These models show that reducing the period of infectiousness by decreasing time to treatment has only a small effect on reducing transmission, implying that passive surveillance needs to be very efficient to prevent resurgence. These data suggest that passive surveillance alone is unlikely to be enough to maintain elimination goals for many “case-finding” NTDs.

## CONSIDERING COSTS AND OTHER RESOURCES

NTD programs also need to carefully consider costs and other resources during decision-making. Therefore, there is a growing need for models to incorporate these factors into projections to inform decision-making more effectively. Economic models can help guide decisions related to new treatments, as in the case of onchocerciasis. For this disease, ongoing concerns that annual MDA of ivermectin may not lead to elimination of parasite transmission in all endemic areas have stressed the importance of considering alternative treatments, particularly the use of moxidectin [19]. However, it is important to consider the economic implications of these alternative strategies. An updated economic assessment of moxidectin- versus ivermectin-based strategies across a range of scenarios (with varied disease prevalence and treatment coverage) suggests that moxidectin-based strategies could not only accelerate progress toward elimination of onchocerciasis transmission but are also likely to reduce programmatic delivery costs compared with ivermectin-based strategies [20].

For helminth infections, including schistosomiasis, there is concern that current diagnostic tools are inadequate for detecting low-intensity infections, highlighting the need for better diagnostics. Transmission models for schistosomiasis, coupled with statistical analysis of *Schistosoma mansoni* egg counts from Burundi, can be used to probe how more sensitive diagnostics can improve decision-making regarding stopping or continuing interventions [21]. These models show that more sensitive diagnostics have a reduced impact on improving health outcomes and are associated with increased costs related to the continuation of MDA interventions unless the stop-MDA threshold is revised. However, if this threshold is set too high, treatment may be stopped too early, resulting in a rebound of infection levels.

Similar trade-offs between additional rounds of treatment and rebounds apply to the design of surveillance strategies for lymphatic filariasis [22]. In this context, modeling can help us to understand how adjusting the threshold used in TASs impacts decisions about the stop of interventions and at what cost. For many settings, a reduction in the threshold increases the probability of elimination, decreases the number of treatment rounds required, and reduces costs. Importantly, however, in certain circumstances (eg, when coverage is lower), lower thresholds can imply an increase in the number of rounds of treatment required to reach that threshold (with increased costs) but help mitigate chronic conditions (such as lymphoedema and hydrocele) and result in longer sustained elimination with fewer future rebounds.

## NEW CHALLENGES AND NEW OPPORTUNITIES

The continued development of increasingly complex NTD models has led to new challenges. While NTD modeling has historically relied on adapting existing models for other diseases, current models are at the forefront of the inference field. As a result, NTD modelers face unique problems, including the need to understand the role of asymptomatic infections in hindering progress to control and eliminate case-finding NTDs [23].

Another significant challenge lies in transitioning from scenario-based global simulations to more local models that can be used to project outcomes and inform decisions at the national and subnational levels. However, to generate meaningful national forecasts, these models require the input of complete and accurate data that reflect local contexts, which are currently limited. One recent example of progress in this area is the advent of new data streams, including from surveys that capture the frequency of never-treated persons in MDA campaigns [11]. Such data, which shed light on previously underrepresented aspects of NTD epidemiology, serve as invaluable input for epidemiological models, heightening their accuracy and potential impact on NTD control, elimination, and surveillance strategies. An additional challenge in this transition to more relevant local models is the need to strengthen in-country capacity and ownership [4], including by training and supporting national modelers and modeling programs.

While limitations regarding locally relevant data persist, scenario-based models built on extensive global datasets will remain important for policy-making, even though they may not always include the exact conditions that reflect individual countries. Furthermore, given that closely tailored analyses are, in general, still reliant on fewer, lower-quality data, they are characterized by high levels of uncertainty, which limit their utility. As data availability and quality increase, local models will become increasingly accurate and relevant, enabling better-informed decisions, including on cost-effectiveness of interventions and surveillance strategies, by incorporating national perspectives on economic analyses. For this, modelers will need to continue to carefully consider what information is needed versus what is available and to work closely with national programs and relevant stakeholders to ensure that models are built on the best possible data and address relevant questions that will aid in making decisions that accelerate achievement of the 2030 targets. Until then, model interpretation and communication of results to different audiences need to be done carefully while acknowledging existing limitations and uncertainty.

## Notes

***Acknowledgments.*** The authors are grateful, as always, to the academic partners, field staff, and neglected tropical diseases (NTDs)–affected populations who generate primary data and invaluable insights that are generously made available to facilitate modeling work.

***Supplement sponsorship.*** This article appears as part of the supplement “New Tools and Nuanced Interventions to Accelerate Achievement of 2030 Roadmap for Neglected Tropical Diseases,” sponsored by funding of Professor T. Déirdre Hollingsworth's research by the Li Ka Shing Foundation at the Big Data Institute, Li Ka Shing Centre for Health Information and Discovery, University of Oxford; and funding of the NTD Modelling Consortium by the Bill & Melinda Gates Foundation (INV-030046).
